# Dual Roles of SIRT7 Inhibition by Oroxylin A Reprogram HSCs Fate: PRMT5 Succinylation-Driven Senescence and Ecto-Calreticulin-Dependent NK Cell Immune Clearance in Liver Fibrosis

**DOI:** 10.34133/research.0808

**Published:** 2025-08-07

**Authors:** Junrui Wang, Haoyuan Tian, Yuanyuan Gao, Xinran Qiu, Zhengyang Bao, Danli Zhao, Feng Zhang, Zili Zhang, Feixia Wang, Shizhong Zheng, Haibo Cheng, Jiangjuan Shao

**Affiliations:** ^1^ Jiangsu Key Laboratory for Pharmacology and Safety Research of Chinese Materia Media, Nanjing University of Chinese Medicine, Nanjing 210023, China.; ^2^ Jiangsu Collaborative Innovation Center of Traditional Chinese Medicine in Prevention and Treatment of Tumor, Nanjing 210023, China.; ^3^ NanTong Health College of Jiangsu Province, Nantong 226000, China.

## Abstract

Activation of hepatic stellate cells (HSCs) represents a central pathological event in liver fibrogenesis, and targeted clearance of activated HSCs is considered to be a promising therapeutic strategy. However, our understanding of the underlying molecular mechanisms is limited. Here, we report that Oroxylin A (OA) inhibited the activation of HSCs by inhibiting the dual roles of Sirtuin 7 (SIRT7). Single-cell transcriptome sequencing analysis and bioinformatics analysis were employed to identify critical pathways, followed by validation through molecular assays including Western blotting, immunofluorescence, and co-immunoprecipitation. In human samples, animal models, and primary cultures, the translational relevance of molecular discoveries was heightened. OA binds to the Gln299 and Asp305 residues of SIRT7, triggering a dual regulatory program in hepatic fibrosis. OA suppresses SIRT7, triggering succinylation-dependent proteasomal degradation of protein arginine methyltransferase 5 (PRMT5). This cascade attenuated symmetric dimethylation of cyclic GMP-AMP synthase (cGAS), thereby activating the cGAS-stimulator of interferon genes (STING) signaling and promoting HSC senescence. Concurrently, OA-elicited SIRT7 inhibition promotes externalized calreticulin (ecto-CRT) expression, thereby enhancing natural killer (NK) cell recognition and targeted elimination of activated HSCs. However, enzymatically dead mutant SIRT7 (H187Y) also suppressed ecto-CRT expression promoted by OA, showing that it is independent of its desuccinylase activity. Our findings reveal a dual regulatory mechanism whereby SIRT7 inhibition by OA coordinates PRMT5 degradation-mediated cellular senescence and ecto-CRT-dependent NK cell immune clearance of HSCs. This work establishes SIRT7 as a pivotal therapeutic target and provides mechanistic insights for developing antifibrotic strategies.

## Introduction

Hepatic fibrosis can be described as an aberrant response to wound healing that is brought on by a chronic, ongoing liver damage [1–3]. There is no known cure for hepatic fibrosis at the moment. In liver injury, activated hepatic stellate cells (HSCs) are the dominant driver of fibrogenesis [1,4,5]. Consequently, it is thought that an efficient therapeutic approach for hepatic fibrosis involves the reduction of activated HSCs and preventing the over-deposition of ECM. Intriguingly, cellular senescence is thought to be a promising approach to eliminate HSCs [6,7]. In recent decades, the application of natural substances to induce cellular senescence has gained acceptance as a promising therapy strategy for treating liver disease [8,9]. Oroxylin A (OA), the active ingredient of *Scutellaria baicalensis*, has an extensive list of pharmacological activities such as anticancer and anticoagulation [10,11]. Our previous study demonstrated that OA activates the cyclic GMP-AMP synthase (cGAS)-stimulator of interferon genes (STING) pathway to induce HSC senescence against hepatic fibrosis [12]. However, the underlying molecular mechanisms remain incompletely elucidated, and the clearance pathway of senescent HSCs has not been fully characterized.

Since precursor proteins lack biological activity, they often require plenty of posttranslational modifications (PTMs) in order to develop into functional mature proteins [13,14]. Common PTMs include DNA/RNA methylation, phosphorylation, and ubiquitination, many of which have been demonstrated to either directly or indirectly control cGAS-STING-mediated innate immune responses [15]. In mammals, protein arginine methyltransferase 5 (PRMT5) catalyzes arginine methylation to influence protein–DNA interactions, suggesting their potential role in modulating the expression of cytosolic DNA sensor cGAS [16]. Notably, PRMT5 activity itself may be regulated by epigenetic modifications [17]. Lysine succinylation (Ksuc), a newly identified PTM, introduces 2 negative charges and a bulky succinyl group to lysine residues, markedly altering protein properties [18,19]. Sirtuin 7 (SIRT7), a sirtuin family member involved in longevity and illness, is a nicotinamide adenine dinucleotide (NAD^+^)-dependent class III histone deacetylase and desuccinylase [17,20–22]. In this study, we reveal for the first time the potential molecular binding mode between OA and desuccinylase SIRT7, and elucidate the mechanistic associations of multiple PTMs (arginine symmetric dimethylation and succinylation) with HSC senescence. However, how senescent HSCs are cleared by the immune system remains incompletely understood.

To preserve hepatic homeostasis, emerging data have shown that both innate and adaptive immune cells could communicate with HSCs and elicit pro- or antifibrotic responses [23,24]. In particular, early activated or senescent HSCs can be directly destroyed by natural killer (NK) cells [25,26], demonstrating an exceptional antifibrotic capacity that may have therapeutic use in fibrotic disorders [27]. However, it is unknown how NK cells identify senescent cells. Sen Santara et al. recently showed that NK cell receptor activating NKp46 is capable of identifying externalized calreticulin (ecto-CRT), which, under conditions of endoplasmic reticulum (ER) stress, travels from the ER to the cell surface. ER stress and NKp46 identification of ecto-CRT in the tumor increase NK cell clearance of senescent tumor cells [28]. This study identifies an important SIRT7-mediated immunosuppressive mechanism in HSCs: SIRT7 reduces ecto-CRT expression by alleviating ER stress independent of its desuccinylase activity, highlighting the functional relevance of the “SIRT7/ER stress/ecto-CRT/NKp46” axis in promoting HSC immune evasion during fibrogenesis. Collectively, in this study, we clarified the mechanisms of OA-triggered HSC senescence in liver fibrosis. The dual-pathway strategy by OA—combining senescence induction and immune clearance—offers a useful approach to fibrosis treatment.

## Results

### OA activates the cGAS-STING pathway by restraining PRMT5 in HSCs

**Fig. 1.**
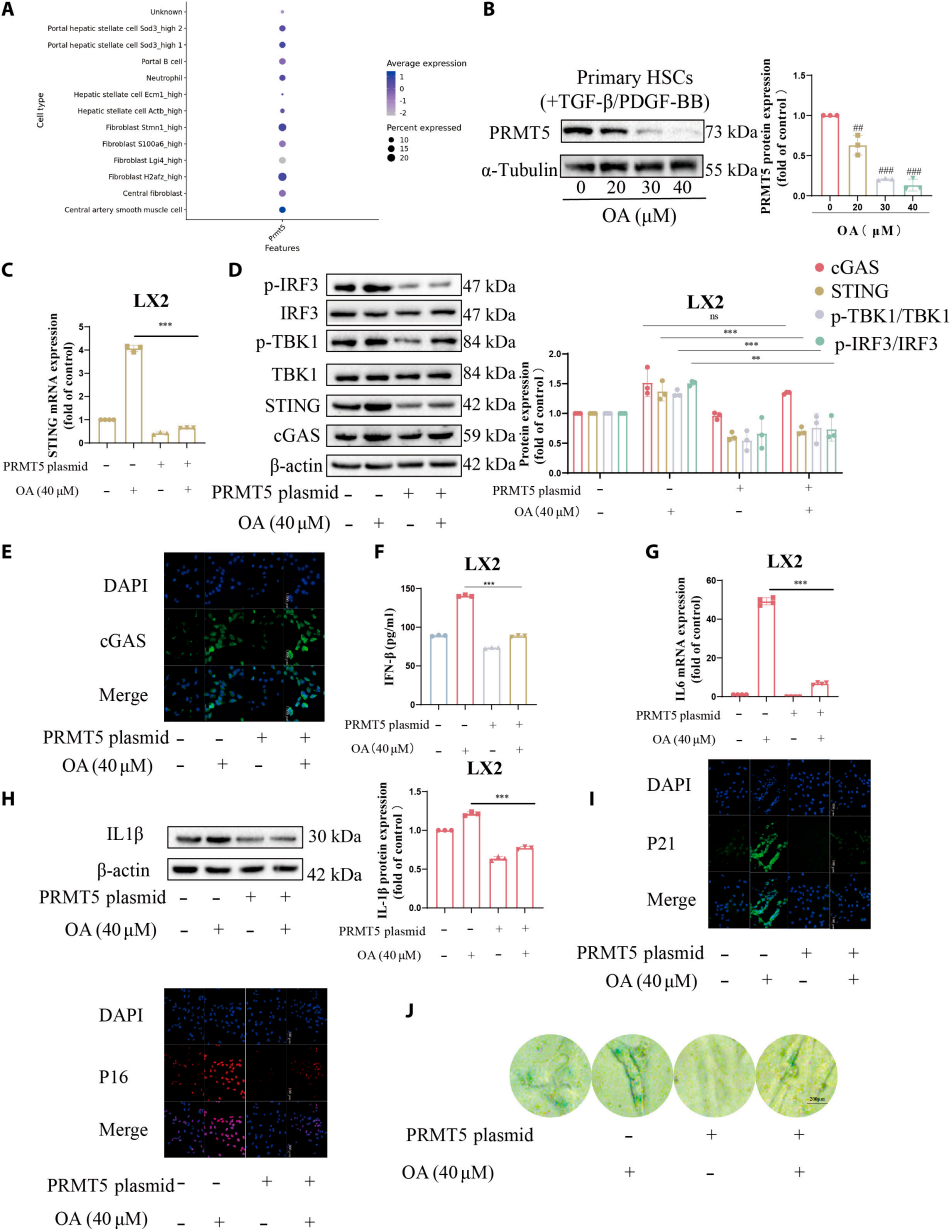
OA activates cGAS-STING pathway by restraining PRMT5 in LX2 cells. LX2 cells were treated with the suggested concentration of OA or PRMT5 plasmid for 24 h. (A) Bioinformatics analysis based on the GSE137720 dataset. Visualize genes’ average expression and the percentage of cells expressing in different cell types (dot plot). (B) Primary mouse hepatic stellate cells were stimulated with a combination of 5 ng/ml TGFβ and 10 ng/ml PDGF-BB for 48 h and cultured for a further 7 days for full activation. Different concentrations of OA were added to intervene for 24 h. The protein expression level of PRMT5 was measured by WB. (C and D) WB and RT-PCR were employed to analyze the expression of the cGAS-STING pathway. (E and I) The levels of cGAS were detected by immunofluorescence. Scale bars are 1,000 μm. The levels of p16 and p21 were detected by immunofluorescence. Scale bars are 100 μm. (F) The IFN-β levels were gauged by the ELISA kit. (G) RT-PCR was applied to measure the mRNA level of IL-1β. (H) WB was used to measure the protein level of IL-6. (J) The percentage of senescent cells was determined with the SA-β-Gal Kit. Scale bars are 200 μm. The data of the graph are displayed as mean ± SD (*n* = 3). Levels of statistical significance are indicated as **P < 0.01, ***P < 0.001 vs. the OA group. ^##^*P* < 0.01, ^###^*P* < 0.001 vs. the control group.

We found that OA induces LX2 cells senescence by stimulating the cGAS-STING pathway [12]. It is reported that the melanoma response to antitumor immunity is defined by PRMT5 modulation of the cGAS/STING and NLRC5 pathways [29]. We used scLiverDB for single-cell transcriptome analysis of the GSE137720_HSC dataset from the Gene Expression Omnibus (GEO) database and visualized PRMT5 gene average expression and the percentage of cells expressing in different cell types (dot plot) [30]. The results showed that compared to other cell types, PRMT5 expression was higher in HSCs (Fig. 1A). Therefore, it is reasonable to speculate that PRMT5 may be involved in OA activating the cGAS-STING pathway. First, we checked the changes of PRMT5 in HSCs after OA treatment. As measured by Western blot (WB) analysis, we found a dose-dependent reduction in the protein expression of PRMT5 (Fig. 1B and Fig. S1A and B). We constructed the PRMT5 overexpression plasmid. As expected, WB demonstrated that PRMT5 overexpression completely impaired the activation of the cGAS-STING pathway induced by OA, but not cGAS expression (Fig. 1C and D). Additionally, immunofluorescence analysis showed a similar result (Fig. 1E). The above results suggest that OA activates the cGAS-STING pathway by reducing the protein level of PRMT5, and this effect may not be achieved by affecting the level of cGAS protein expression. Remarkably, we discovered that PRMT5 overexpression dramatically lowered the level of interferon-β (IFN-β) (Fig. 1F). Next, we explored whether the up-regulation of PRMT5 affects HSC senescence. As anticipated, WB and PCR results showed that overexpression of PRMT5 attenuated the elevation of multiple senescence-associated secretory phenotype (SASP) expression mediated by OA, like interleukin-1β (IL-1β) and IL-6 (Fig. 1G and H). Meanwhile, PRMT5 overexpression markedly blocked the rise in p16 and p21 expression and the high activity of β-galactosidase induced by OA (Fig. 1I and J). Taken together, the results imply that OA activates the cGAS-STING pathway by reducing PRMT5 protein expression to induce HSC senescence.

### OA inhibits PRMT5-mediated dimethylation of cGAS

**Fig. 2.**
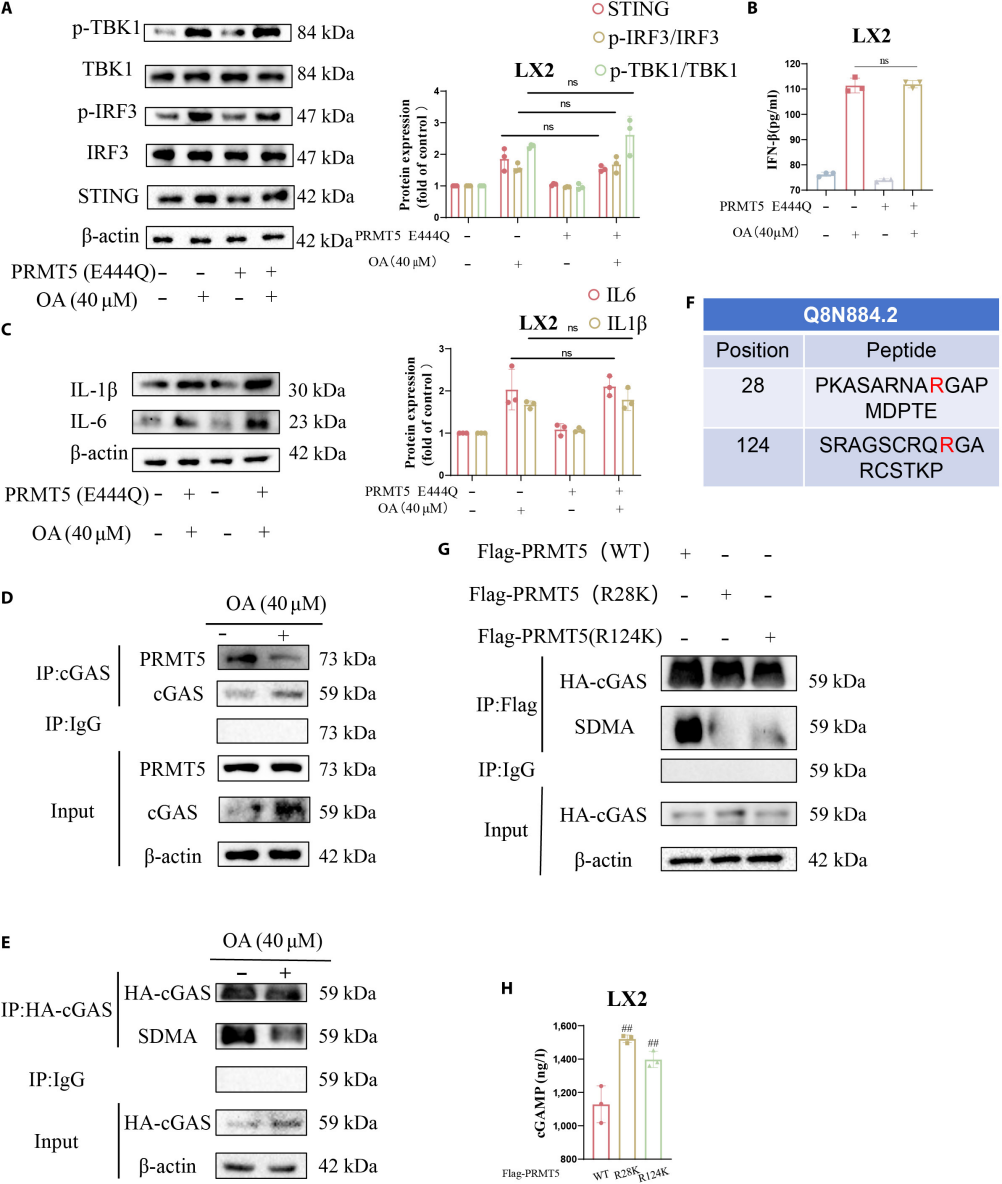
OA inhibits PRMT5-mediated dimethylation of cGAS in LX2 cells. (A) Protein levels of cGAS-STING were measured by WB. (B) The IFN-β level was gauged by the ELISA kit. (C) WB was utilized to measure the protein levels of IL-6 and IL-1β. (D) IP analysis showed the interaction between cGAS and PRMT5. (E) The methylation level of HA-tagged cGAS was gauged by IP analysis. (F) GPS-MSP was used to predict symmetrical di-methylation sites of cGAS. (G) The methylation level of HA-tagged cGAS was shown by IP analysis. (H) The cGAMP level was detected by the ELISA kit. Levels of statistical significance are indicated as ns, not significant vs. OA group. ^##^*P* < 0.01 vs. control group.

We have demonstrated that OA triggers the cGAS-STING pathway by restraining PRMT5, and this effect may not be achieved by changing the expression level of cGAS. The main type II arginine methyltransferase that methylates arginine residues symmetrically is designated as PRMT5. Therefore, we hypothesized that PRMT5 inhibited OA from activating the cGAS-STING pathway by regulating arginine-symmetric dimethylation of cGAS in LX2 cells. We transfected the enzymatically dead mutant PRMT5 (E444Q) into LX2 cells and then treated them with OA. Our data showed that OA markedly enhanced STING expression in LX2 cells and triggered phosphorylation of TBK1 and IRF3, an effect not reversed by PRMT5 (E444Q) (Fig. 2A). The results of interleukin and IFN-β expression were likewise consistent (Fig. 2B and C). According to the aforementioned findings, that PRMT5 inhibits OA from activating cGAS-STING pathway may depend on its enzymatic activity.

Next, we found that OA attenuates the binding of PRMT5 to cGAS while reducing the level of arginine-symmetric dimethylation of cGAS (Fig. 2D and E and Fig. S1C). Therefore, we further tried to screen the arginine symmetric dimethylation sites on cGAS by Group-based Prediction System–Methyl-group Specific Predictor (GPS-MSP) [31], which can predict mono-, di-, and tri-methylation types for specific lysines, as well as mono- and symmetric and asymmetric dimethylation types for specific arginines, and identified 2 sites: R28 and R124 (Fig. 2F). We created mutants by changing each arginine to a lysine residue (R28K and R124K) in order to further define the exact residues for symmetric dimethylation of cGAS. The dimethylation of cGAS caused by PRMT5 was markedly reduced when the arginine at Arg28 and Arg124 was substituted with lysine (R28K and R124K), according to the immunoprecipitation (IP) experiment, which indicated that the Arg28 and Arg124 residue are the catalytic sites of PRMT5 (Fig. 2G). Further studies showed that both cGAS (R28K) and cGAS (R124K) plasmids enhanced cGAMP expression (Fig. 2H). PRMT5 catalyzes the dimethylation of cGAS at residues Arg 28 and Arg124 in LX2 cells. Arginine methylation of cGAS may affect cGAS catalyzing the synthesis of cGAMP. In summary, our data validated that in LX2 cells, OA blocking PRMT5-mediated dimethylation of cGAS stimulates the cGAS-STING pathway.

### OA inhibits SIRT7 from desuccinylating PRMT5

**Fig. 3.**
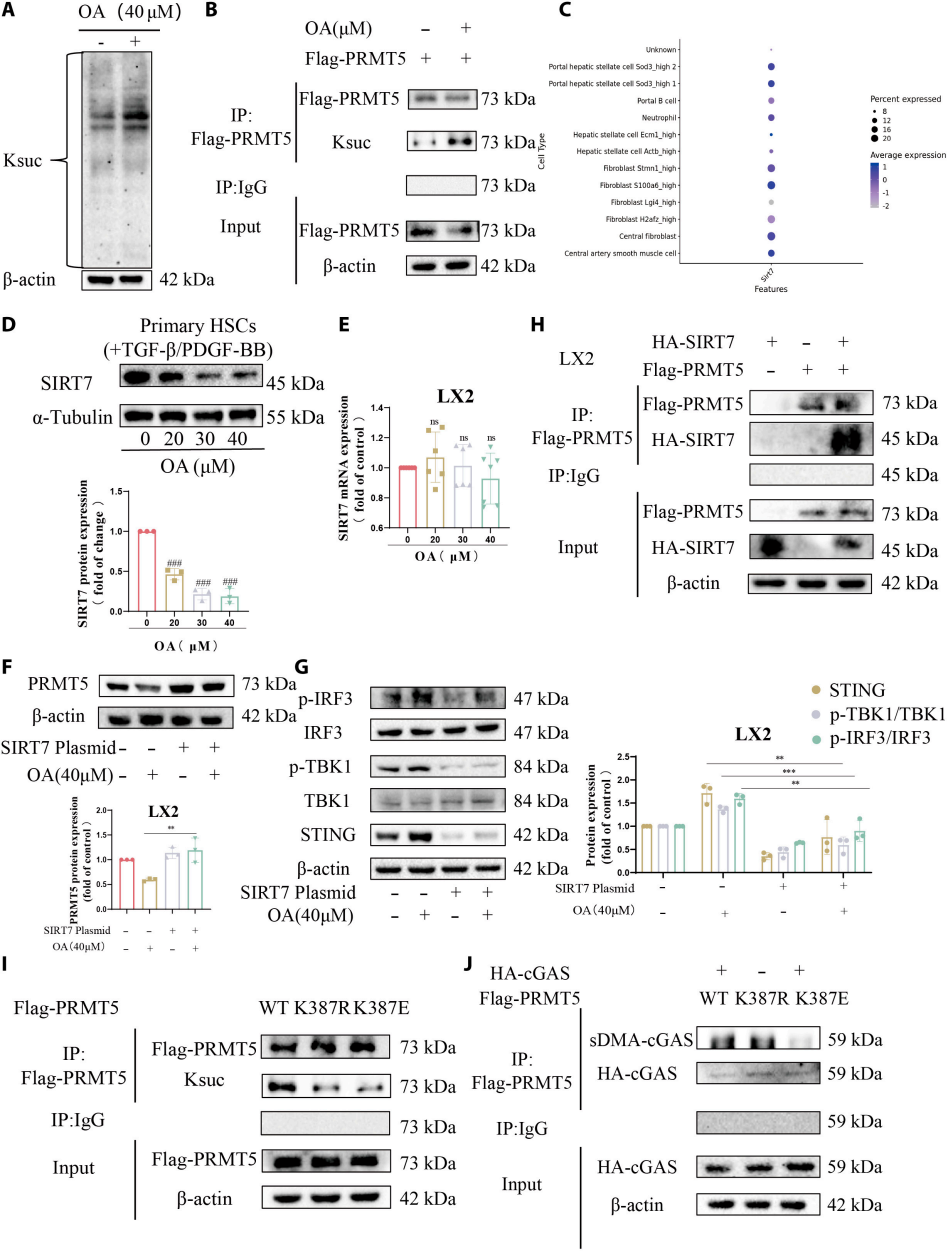
OA promotes PRMT5 protein degrading by inhibiting SIRT7 from desuccinylating PRMT5 in LX cells. (A) Succinylation level was tested by WB. (B) Succinylation level of PRMT5 was tested by IP analysis treated with OA or DMSO. The succinylation level of PRMT5 was shown by IP analysis. (C) Bioinformatics analysis based on the GSE137720 dataset. Visualize genes’ average expression and the percentage of cells expressing in different cell types (dot plot). (D) Primary mouse hepatic stellate cells were stimulated with a combination of 5 ng/ml TGFβ and 10 ng/ml PDGF-BB for 48 h and cultured for a further 7 days for full activation. Different concentrations of OA were added to intervene for 24 h. The protein expression level of SIRT7 was measured by WB. (E) SIRT7 mRNA expression was gauged by RT-PCR. (F) Protein level of PRMT5 was measured by WB. (G) Protein levels of the cGAS-STING pathway were measured by WB. (H) IP analysis of the interaction between HA-SIRFT7 and Flag-PRMT5. (I) The succinylation level of PRMT5 or PRMT5 mutants was shown by IP analysis. (J) The methylation levels of HA-tagged cGAS were gauged by IP analysis. Levels of statistical significance are indicated as **P < 0.01, ***P < 0.001 vs. OA group. ^###^*P* < 0.001; ns, not significant vs. control group.

Ksuc is a newly discovered PTM of lysine residues that triggers more changes in protein properties [13,19]. We found an interesting phenomenon that the overall succinylation level increased in LX2 cells after OA treatment (Fig. 3A). Therefore, we speculated that OA may regulate the succinylation of PRMT5. Subsequent IP experiments demonstrated that OA increased the succinylation of PRMT5 but had no effect on its acetylation level (Fig. 3B and Fig. S1D). It has been reported that SIRT7, which desuccinylates PRMT5, inhibits the ubiquitinated degradation of PRMT5 [17]. Single-cell transcriptome analysis of the GSE137720_HSC dataset from the GEO database results showed that SIRT7 expression was higher in HSCs compared to other cell types (Fig. 3C) [30]. In a CCl_4_-induced mouse model of liver fibrosis, SIRT7 expression in activated HSCs increased with disease progression (Fig. S1E). So how does SIRT7 alter in primary mouse HSCs treated with OA? In light of this uncertainty, using the WB experiment, it is surprising that the protein level of SIRT7 decreased dose-dependently (Fig. 3D and Fig. S1F and G). However, OA did not cut the mRNA level of SIRT7 (Fig. 3E). Therefore, we hypothesized that OA may promote PRMT5 protein degrading by inhibiting SIRT7 from desuccinylating PRMT5 and thereby activate the cGAS-STING pathway.

We constructed the SIRT7 overexpression plasmid. We found that OA substantially suppressed PRMT5 expression in LX2 cells, while the overexpression of SIRT7 restored PRMT5 expression (Fig. 3F). Next, WB showed that OA substantially enhanced the protein levels linked to the cGAS-STING pathway (Fig. 3G). Besides, we confirmed that OA has an important anti-liver fibrosis effect, while the overexpression of SIRT7 reversed the effect (Fig. S2A to C). At the same time, compared with the SIRT7-knockdown group, OA treatment in SIRT7-knockdown cells did not further suppress the expression of PRMT5 and liver fibrosis markers (Fig. S2D and E). These results demonstrate that SIRT7 inhibition is necessary for OA’s antifibrotic action. The co-IP experiments demonstrated the interaction between SIRT7 and PRMT5 (Fig. 3H). The above experiments suggest that OA may reduce the expression of PRMT5 and liver fibrosis-related indicators by inhibiting SIRT7 from interacting with PRMT5 and thereby stimulate the cGAS-STING pathway.

Next, we wanted to probe the succinylation sites of PRMT5 in LX2 cells. A previous study has suggested that lysine residue 387 (K387) may be the succinylation site for PRMT5 [17]. Then, we generated 2 mutants: K387E (lysine to glutamate), which mimics the negatively charged succinyllysine alteration, and K387R (lysine to arginine), which mimics the desuccinylated state. K387 may be a succinylation site of PRMT5, as succinylation of PRMT5 mutants (K387R and K387E) markedly decreased (Fig. 3I), while their acetylation levels remained unchanged (Fig. S2F). While PRMT5 K387E displayed the opposite effect, IP experiments implied that PRMT5 K387R boosted the symmetric dimethylation of cGAS (Fig. 3J). Interestingly, the PRMT5 mutants also reduced the binding of PRMT5 to SIRT7. K387 may be the binding site of PRMT5 to SIRT7 (Fig. S2F). It implies that succinylation of PRMT5 K387 impaired the symmetric dimethylation level of cGAS by PRMT5.

### Gln299 and Asp305 are crucial for the binding of SIRT7 to OA in LX2 cells

**Fig. 4.**
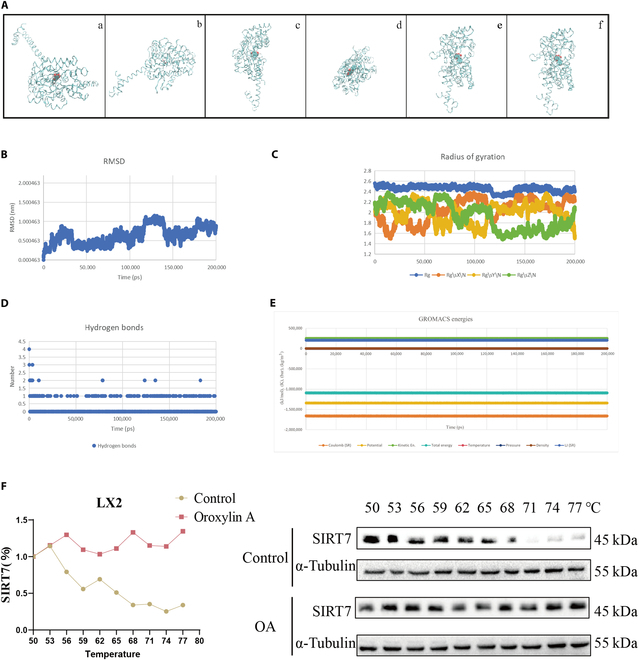
The OA–SIRT7 complex shows positive stability. (A) OA–SIRT7 complexes at various time breaks respectively are captured in panels (a) to (e). Panel (f) is the representative structure generated by cluster analysis of 200 snapshots. (B) RMSD fluctuation of OA–SIRT7 complexes. (C) Radius of gyration fluctuation of OA–SIRT7 complexes. (D) Hydrogen bond simulation. The time scale is represented by the horizontal axis, while the quantity of hydrous bonds is indicated by the vertical axis. Different data regarding density, pressure, energy, and other variables during the simulation process. (E) A compilation of data about energy, pressure, density, and further simulation-related changes. (F) After incubating LX2 cells with or without OA (40 μM) for 2 h, the cells were removed and exposed to the CETSA test.

We used molecular dynamics simulations to examine the interaction between OA and the crystal structure of SIRT7 to understand the mechanism behind the cut of SIRT7 protein production by OA. The 200-ns trajectory analysis yields 200 snapshots. A snapshot is created every 1 ns. For visualization, the simulation system’s trajectories of 0, 50, 100, 150, and 200 ns were chosen (Fig. 4A). The root mean square deviation (RMSD) values in the OA–SIRT7 complex change minimally between 0 and 1.5 nm in the 200-ns MD simulations (Fig. 4B), suggesting that SIRT7 coupled with OA rather well. If the protein remained stable, the radius of gyration—a crucial factor in complex stability—would, on average, level out. The evidence of OA–SIRT7 complex stability can be found in Fig. 4C. The OA–SIRT7 complex exhibited an average of 1 to 2 hydrogen bonds established during the simulation, indicating conformational stability (Fig. 4D). A compilation of data about energy, pressure, density, and further simulation-related changes is listed in Fig. 4E and Table S2. This information can be used to show the equilibrium state. The direct interaction between OA and SIRT7 was additionally verified by cellular thermal shift assay (CETSA) (Fig. 4F). The OA–SIRT7 combination, therefore, shows encouraging stability.

**Fig. 5.**
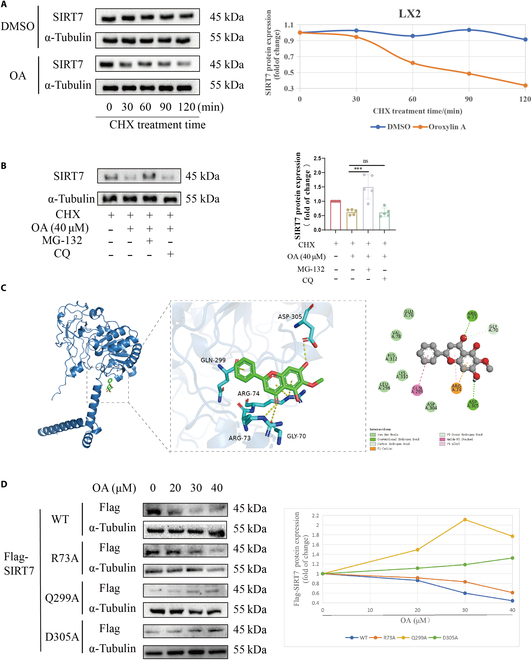
Gln299 and Asp305 are essential in OA-mediated SIRT7 degradation. (A) The expression of SIRT7 protein was gauged by WB. (B) The expression of SIRT7 protein was detected by WB. (C) Molecular docking analysis of the potential binding between OA and SIRT7. (D) Comparison of exogenous SIRT7-WT/SIRT7-R73A/SIRT7-Q299A/ SIRT7-D305A expressions in LX2 cells treated with OA (0, 20, 30, and 40 μM) for 24 h. Levels of statistical significance are indicated as ***P < 0.001; ns, not significant vs. the OA group.

Next, we explored how OA reduced SIRT7. We hypothesized that OA might be involved in the degradation of SIRT7 protein because prior experimental results demonstrated that OA had no impact on the mRNA level of SIRT7 but lowered SIRT7 protein (Fig. 3D and E). We investigated how OA affected the stability of SIRT7 protein when treated with cycloheximide (CHX). The findings demonstrated that, following CHX treatment, OA considerably accelerated the rate at which SIRT7 protein expression decreased in LX2 cells (Fig. 5A). Autophagy and ubiquitin are the 2 primary mechanisms of protein degradation, as is well known. We employed the autophagy inhibitor chloroquine (CQ), the selective 26S proteome inhibitor MG-132, and the protein biosynthesis inhibitor CHX to explore whether OA decreases SIRT7 expression by promoting the ubiquitination or autophagy of SIRT7. We observed that OA cut the protein expression of SIRT7, while MG-132 restored it. Nevertheless, we discovered that the autophagy inhibitor CQ did not reverse the SIRT7 decrease in LX2 cells (Fig. 5B). These results showed that OA decreases the protein level of SIRT7 by regulating the degradation by ubiquitination of SIRT7.

Then, we found several critical directional interactions between the OA and the SIRT7; the Asp305 and Arg73 residues on SIRT7 protein form a hydrogen bond interaction with OA. The Gln299 residue forms a hydrophobic interaction with OA and is located in the catalytic core of the SIRT7 protein (Fig. 5C). To identify the key amino acids of SIRT7 implicated in interactions with OA, the stability of wild-type SIRT7, SIRT7-R73A, SIRT7-Q299A, and SIRT7-D305A mutant proteins following OA treatment was evaluated. Compared to SIRT7-Q299A and SIRT7-D305A, treating transfected LX2 cells with OA reduced the SIRT7-WT and SIRT7-R73A protein levels dose-dependently (Fig. 5D), suggesting that Gln299 and Asp305 play a potential role in OA-mediated SIRT7 degradation.

### SIRT7 blockade by OA reduces liver fibrogenesis in vivo

**Fig. 6.**
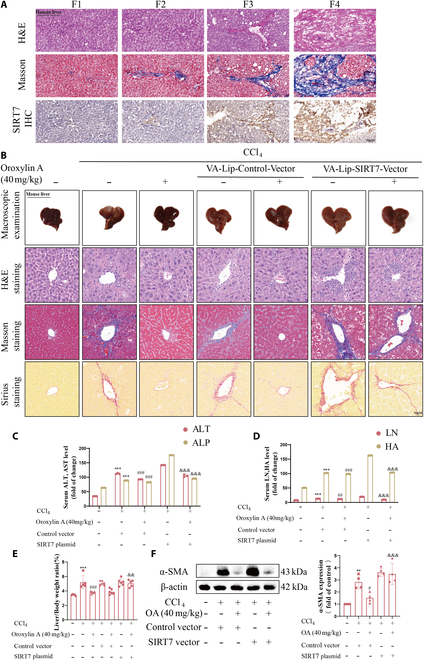
Increasing SIRT7 expression in vivo will diminish the antifibrosis effect of OA. (A) The liver fibrosis stage was assessed by Metavir criteria. For histopathological study, human liver samples (F0/1, 3; F2, 3; F3, 4; F4, 5) were stained with H&E Masson and IHC for SIRT7. Representative images were shown. Scale bar: 50 μm. *n* = 3 per group. (B) The livers were taken out from the animals after they were killed and photographed so that the shape of the liver could be seen. (C and D) Items of liver fiber (HA and LN) in serum and indexes of liver injury (ALT and ALP) were detected. (E) Determine the ratio of liver to body weight. *n* = 6 per group. (F) The expression of α-SMA protein in liver tissues was detected by WB. **P < 0.01; ***P < 0.001, compared with the control group; ^#^*P* < 0.05, ^##^*P* < 0.01, ^###^*P* < 0.001, compared with the CCl_4_ group; ^&&^*P* < 0.01, ^&&&^*P* < 0.001, compared with the CCl_4_ +OA+vector group).

To further investigate the relationship between SIRT7 and the pathological progression of hepatic fibrosis, we analyzed the expression of SIRT7 in clinical samples of human hepatic fibrosis (Fig. 6A). Initially, hematoxylin and eosin (H&E) stains and Masson stains showed the fibrotic progression (Fig. 6B). Immunohistochemical (IHC) analysis revealed that SIRT7 expression demonstrated a progressive upward trend with advancing disease stages. Vitamin A-coupled liposomes carrying SIRT7 plasmid (VA-LipSIRT7-plasmid) were created to raise HSC-specific SIRT7 expression. Macroscopically observing the liver morphology, the fibrosis pathology of the CCl_4_ group was distinctive from the control group, whereas treating with OA weakened the hepatic fibrosis induced by CCl_4_. As expected, OA did not improve hepatic fibrosis when pretreatment with VA-Lip-SIRT7 plasmid was administered (Fig. 6B). Additionally, Masson staining, Sirius red staining, and H&E staining showed that OA treatment greatly trimmed down collagen deposition in the central vein and VA-lip-SIRT7 plasmid reversed this effect (Fig. 6B). By examining alanine transaminase (ALT) and alkaline phosphatase (ALP) in serum, markers of liver injury, we observed that VA-Lip-SIRT7 plasmid trimmed down the beneficial effects of OA (Fig. 6C). Simultaneously, the expression of hepatic fibrosis markers hyaluronic acid (HA) and laminin (LN) were also altered by the SIRT7 plasmid (Fig. 6D). Through liver weight ratio calculations, the liver weight ratio of mice in the CCl_4_+OA+VA-Lip-SIRT7 plasmid group was considerably higher than that of the OA group (Fig. 6E). The levels of α-smooth muscle actin (α-SMA) were markedly higher than mice in the OA+SIRT7 group compared to mice treated with that of the OA group alone (Fig. 6F).

Immunofluorescence analysis showed that compared with mice in the OA group, the level of activated HSC markers α-SMA was considerably higher in mice of the CCl_4_+OA+VA-Lip-SIRT7 plasmid group (Fig. S3A). Meanwhile, cGAS and STING had a higher abundance in activated HSCs of OA-treated mice compared with controls, and the SIRT7 plasmid reversed the enhancement in STING expression induced by OA, rather than cGAS expression (Fig. S3B and C). This is further evidence that PRMT5 may inhibit OA from activating the cGAS-STING pathway by regulating arginine-symmetric dimethylation of cGAS in HSCs. In addition, VA-Lip-SIRT7 plasmid markedly reversed the suppression of PRMT5 in activated HSCs by OA (Fig. S3D). In conclusion, these results showed that OA inhibiting SIRT7 promotes succinylation of PRMT5 and its degradation and thereby attenuates symmetric dimethylation of cGAS in HSCs, thus triggering the cGAS-STING pathway.

### SIRT7 blockade by OA promotes CRT exposure in HSCs, independent of the desuccinylase activity of SIRT7

**Fig. 7.**
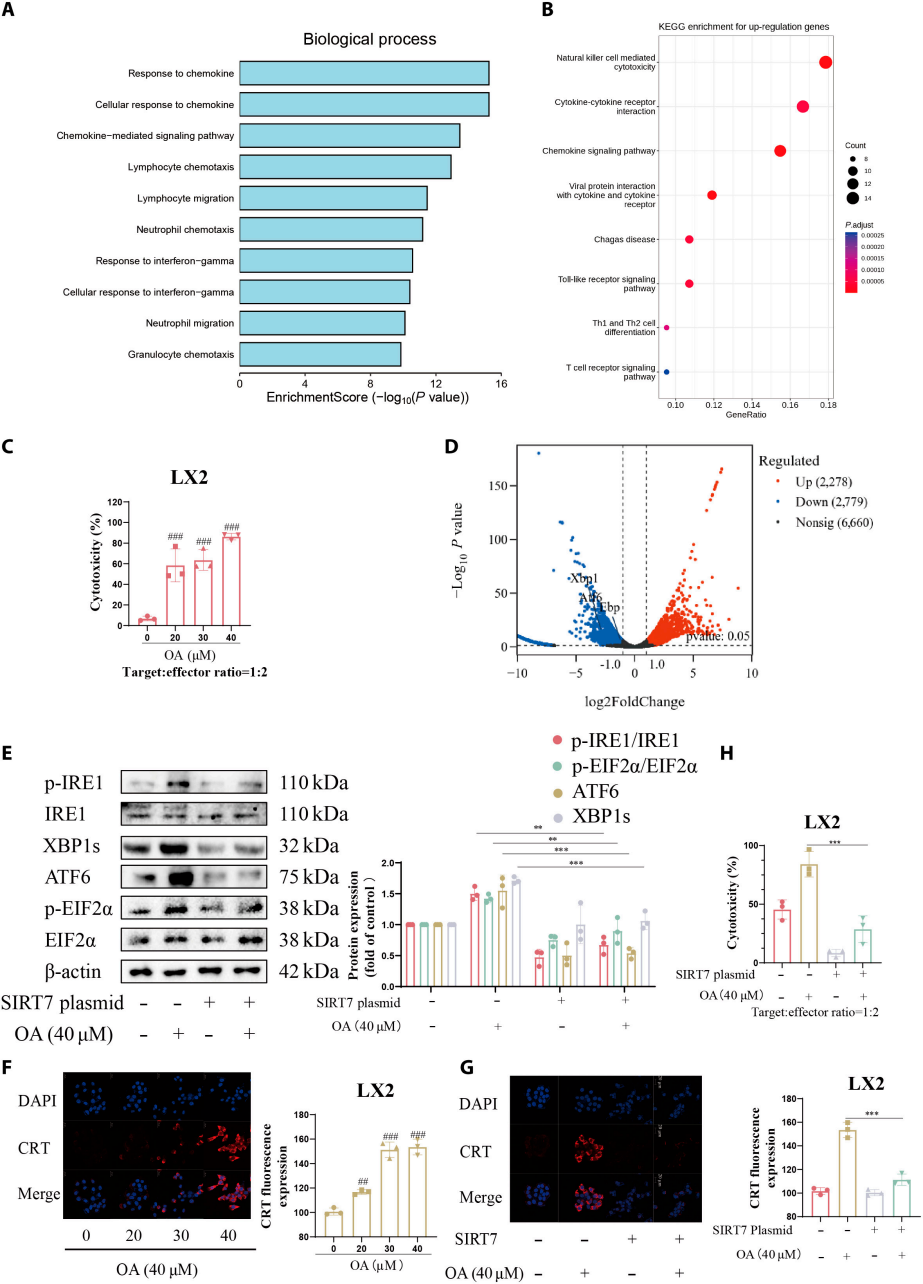
SIRT7 blockade by OA promotes CRT externalization, thus increasing NK cell recognizing and killing HSCs. (A) Bioinformatics analysis based on the GSE84044 dataset. Gene set enrichment analysis (GSEA) was used to further reveal the expression of differential genes in the progression of hepatic fibrosis. (B) Bioinformatics analysis based on the GSE136103 dataset. Statistical enrichment of differentially expressed genes in KEGG pathways. (C) The cytotoxicity was calculated when the target-to-effector cell ratio is 1:2. (D) Volcano map of significant differentially expressed genes. (E) WB analyzed the expression of ERS-associated proteins in HSCs. The data are shown as mean ± SD (*n* = 3). (F) The level of ecto-CRT was detected by immunofluorescence and quantified with ImageJ software. Representative photographs were shown. Scale bars are 20 μm. (G) The level of ecto-CRT was detected by immunofluorescence. (H) The cytotoxicity was calculated when the target-to-effector cell ratio is 1:2. The data are shown as mean ± SD (*n* = 3). **P < 0.01, ***P < 0.001 vs. the OA group. ^##^*P* < 0.01, ^###^*P* < 0.001 vs. the control group.

**Fig. 8.**
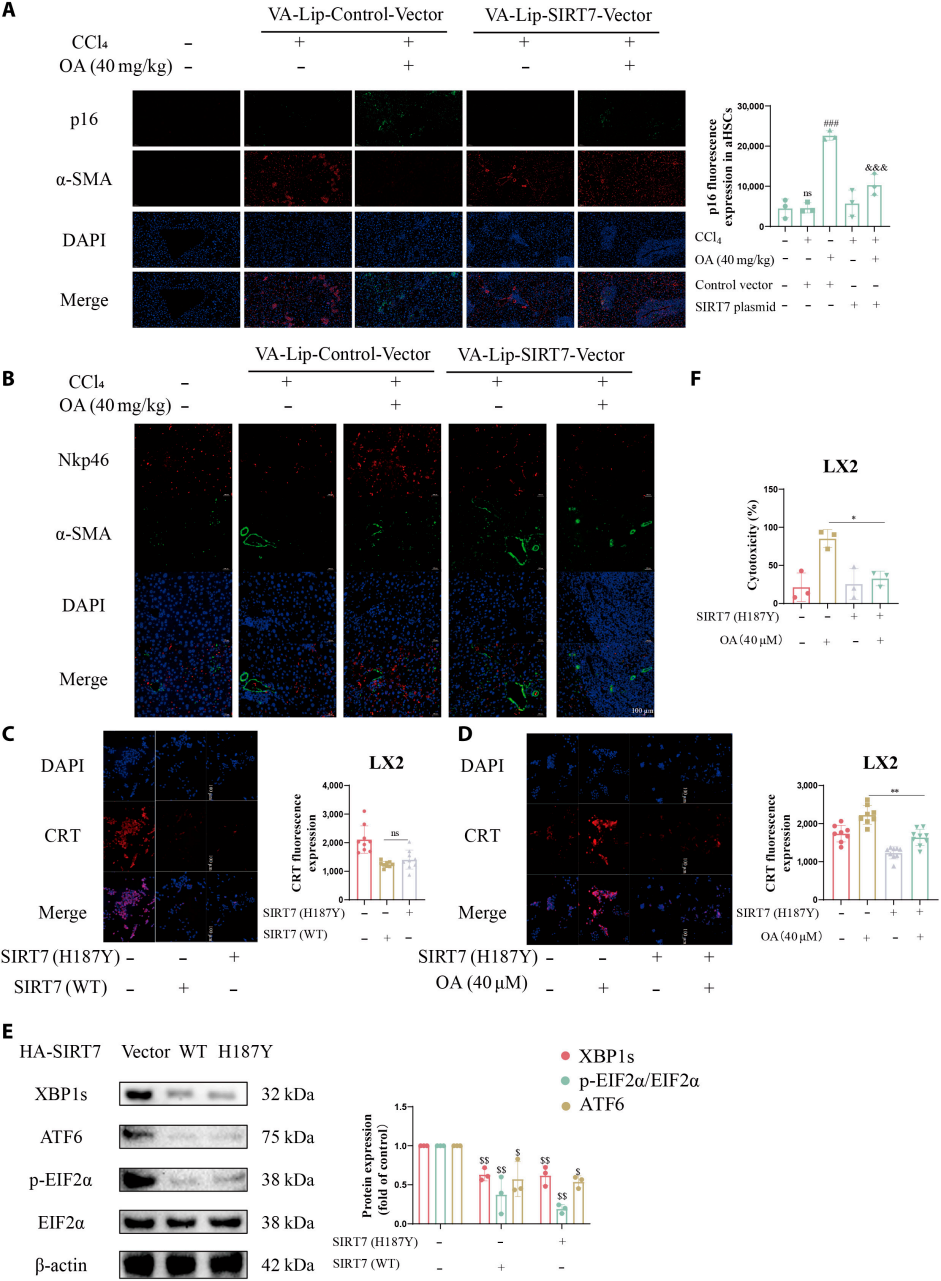
SIRT7 blockade by OA promotes CRT exposure in HSCs, independent of the desuccinylase activity of SIRT7. (A) Immunofluorescence screening was employed to detect the protein expression and colocalization of α-SMA and p16. The scale bar is 50 μm. (B) Utilizing immunofluorescence screening, the expression of Nkp46 and α-SMA proteins was identified. Antibodies against NKp46 were utilized in immunofluorescence staining of liver slices in order to precisely label NK cells. The HSCs were selectively stained with an antibody against α-SMA. The scale bar is 100 μm. (C) The level of ecto-CRT was detected by immunofluorescence and quantified with ImageJ software. Representative photographs were shown. Scale bars are 100 μm. (D) The level of ecto-CRT was detected by immunofluorescence and quantified with ImageJ software. Representative photographs were shown. Scale bars are 100 μm. (E) WB analyzed the expression of ERS-associated proteins in HSCs. (F) The cytotoxicity was calculated when the target-to-effector cell ratio is 1:2. The data are shown as mean ± SD (*n* = 3). ns, not significant, ^$^*P* < 0.05, ^$$^*P* < 0.01 vs. the control group; *P < 0.05, **P < 0.01, compared with the OA group; ^###^*P* < 0.001, compared with the CCl_4_+vector group; ^&&&^*P* < 0.001, compared with the CCl_4_+OA+vector group).

Bioinformatic analysis revealed that the genes linked to response to chemokine changed significantly as hepatic fibrosis developed (Fig. 7A). Chemokines are chemotactic cytokines that control the migration of immune cells [32]. In order to preserve liver homeostasis, both innate and adaptive immune cells could communicate with HSCs and elicit pro- or antifibrotic responses [1]. In particular, early activated or senescent HSCs can be directly destroyed by NK cells [7]. Meanwhile, Kyoto Encyclopedia of Genes and Genomes (KEGG) enrichment analysis of up-regulated differentially expressed genes (UP DEGs) in NK cells of the GSE136103 dataset from the GEO database showed that genes related to NK cell-mediated cytotoxicity were the most significantly enriched in the progression of hepatic fibrosis (Fig. 7B), demonstrating an exceptional antifibrotic capacity that may have therapeutic use in fibrotic disorders [33]. We found an interesting phenomenon wherein NK cells were capable of killing OA-treated HSCs, and their killing was enhanced in a dose-dependent manner (Fig. 7C), without substantial killing of untreated HSCs. Although NK cell recognizing HSCs helps inhibit hepatic fibrosis [34], how OA regulates NK cell killing HSCs remains unknown. A recent study found that the NK cell receptor NKp46 identifies ecto-CRT on ER-stressed senescent cells. Interestingly, the transcriptomics results indicated a decline in the levels of ER stress-related genes in the CCl_4_ group (Fig. 7D). In light of the aforementioned experimental results, we boldly speculate that OA may promote CRT exposure by regulating ER stress by inhibiting SIRT7 in HSCs and thus enhance NK cell recognizing and killing HSCs. First, we performed SIRT7 overexpression experiments in HSCs and observed that SIRT7 overexpression reversed up-regulation of ER stress-related proteins including p-IRE1, XBP1s, p-EIF2α, and ATF6 induced by OA (Fig. 7E). Confocal experiments demonstrated that OA dose-dependently enhanced ecto-CRT on the surface of HSCs, which was reversed by overexpression of SIRT7 in HSCs (Fig. 7F and G). Besides, we found that SIRT7 overexpression in HSCs reversed the OA-induced NK cell killing increase (Fig. 7H). In vivo experiment confirmed that the level of senescence in activated HSCs and the number of NK cells surrounding the HSC increased after OA treatment and the effect was reversed by overexpression of SIRT7 in HSCs (Fig. 8A and B). These results verified our hypothesis.

Given the desuccinylase activity of SIRT7, we wanted to understand whether SIRT7 inhibition of HSC CRT exposure depends on its enzymatic activity. We transfected the enzymatically dead mutant SIRT7 (H187Y) into LX2 cells and then treated them with OA. Compared to SIRT7 WT, SIRT7 H187Y also suppressed ER stress and CRT exposure (Fig. 8C to E) in LX2 cells. Consistent with this, SIRT7 H187Y (Fig. 8F) inhibited HSC killing by NK cells. Therefore, we concluded that SIRT7 inhibits HSC CRT externalization independent of its enzymatic activity.

## Discussion

Our previous study found that *S. baicalensis*-derived OA is able to induce HSC senescence by stimulating the cGAS-STING pathway, thus inhibiting hepatic fibrosis [12]. However, how OA activates the cGAS-STING pathway remains incompletely obscure. Our experimental outcomes showed that OA dose-dependently reduced the protein expression of PRMT5, while overexpression of PRMT5 reversed the elevation of cGAS-STING pathway expression by OA (Fig. 1). Interestingly, the enzymatic death mutant (E444Q) could not reverse the elevation of cGAS-STING pathway expression, suggesting that PRMT5 inhibiting OA from activating the cGAS-STING pathway may depend on its enzymatic activity (Fig. 2). Subsequently, we further tried to screen the arginine symmetric dimethylation sites on cGAS. Our investigation proved that the R28 and R124 residues are the direct catalyzing site by PRMT5, and the cGAS (R28K and R124K) mutants attenuated the dimethylation of cGAS and eliminated the PRMT5-mediated inhibition of cGAMP synthesis, a key intermediate for cGAS synthesis (Fig. 2). However, Ma et al. [35] reported that PRMT5 catalyzes cGAS only at the R124 residue. These differences make sense because of the varied modified targets and mechanism that PRMT5 might have in differently originating cells through distinct bioprocesses.

Ksuc is a newly discovered PTM of lysine residues that triggers more changes in protein properties [19]. We found that OA increased the succinylation of PRMT5. Our experimental outcomes showed that OA dose-dependently reduced the protein expression of SIRT7, while overexpression of desuccinylase SIRT7 reversed the down-regulation of PRMT5 expression and up-regulation of cGAS-STING pathway expression by OA (Fig. 3). Subsequently, we demonstrated the direct binding of SIRT7 to PRMT5. We further tried to screen the succinylation sites on PRMT5. It is possible that K387 is a succinylation site of PRMT5 because we were unable to detect the succinylation of Flag-tagged PRMT5 mutants (K387R and K387E) in LX2 cells. IP experiments showed that PRMT5 K387R substantially raised the symmetric dimethylation of cGAS, but PRMT5 K387E showed the opposite effect, suggesting that K387, the succinylation site of PRMT5, impaired the dimethylation level of cGAS by PRMT5 (Fig. 3).

Our study suggested that the desuccinylation of PRMT5 by SIRT7 raises the symmetric dimethylation of cGAS. There appeared to be disagreement in recent publications regarding the role of SIRT7 in the PRMT5 methyltransferase activity. It has been shown that the WDR77/PRMT5 transmethylase complex activity is decreased when WDR77 is deacetylated by SIRT7[36]. In contrast, a recent study reported that dependent on SIRT7-mediated PRMT5 K387 desuccinylation in tumors, PRMT5 contributes lipid metabolic reprogramming, tumor development, and metastasis [17], which is consistent with our study. The contradictory role may be due to different mechanisms in different biological processes and needs further exploration. Meanwhile, a recent study showed that SIRT7 has a beneficial effect on hepatic fibrosis. This seems to be inconsistent with our findings. However, Ding et al. [37] studied the effect of SIRT7 in myeloid cells, so this difference is also understood. This also awaits further study.

In our study, OA reduced SIRT7 protein, which is caused by the proteasome degradation pathway (Figs. 4 and 5). We found a number of possible active sites for investigating the interaction between OA and SIRT7 using molecular docking techniques. Compared with Flag-WT protein, Flag-Q299A protein and Flag-D305A protein are more resistant to destruction by OA because of their weaker interactions with OA. When considered collectively, we find that the Gln299 and Asp305 sites are essential to the stability of SIRT7 when it interacts with OA (Figs. 4 and 5).

NK cells exhibit a unique antifibrotic property by eliminating senescent or early activated HSCs directly [7], which might be valuable as a treatment for fibrotic illnesses [33]. Ecto-CRT is a newly discovered ligand for the NK cell receptor Nkp46. For the first time, our research demonstrated that OA is able to increase CRT on the cell surface by inhibiting SIRT7 in HSCs, thus enhancing senescent HSC recognition and killing by NK cells. This enhancement is independent of the desuccinylase activity of SIRT7 (Fig. 7 and 8). MICA and ULBP2 are ligands of the NK cell receptor NKG2D, which is why NK cells can destroy senescent HSCs produced by curcumin, according to a prior study [38]. This is logical enough, because both NKp46 and NKG2D might affect NK cell killing of senescent cells. That NK cells kill senescent cells is boosted by ER stress and NKp46 identification of ecto-CRT [28]. Sadly, our research could not pinpoint a particular ER stress pathway that is necessary for CRT externalization, indicating that more investigation is necessary to find the exact mechanism by which ER stress results in CRT exposure. The study of Zhang et al. [39] reported the relationship between ER stress and cell senescence, which does not conflict with our study, but in-depth studies require further exploration.

**Fig. 9.**
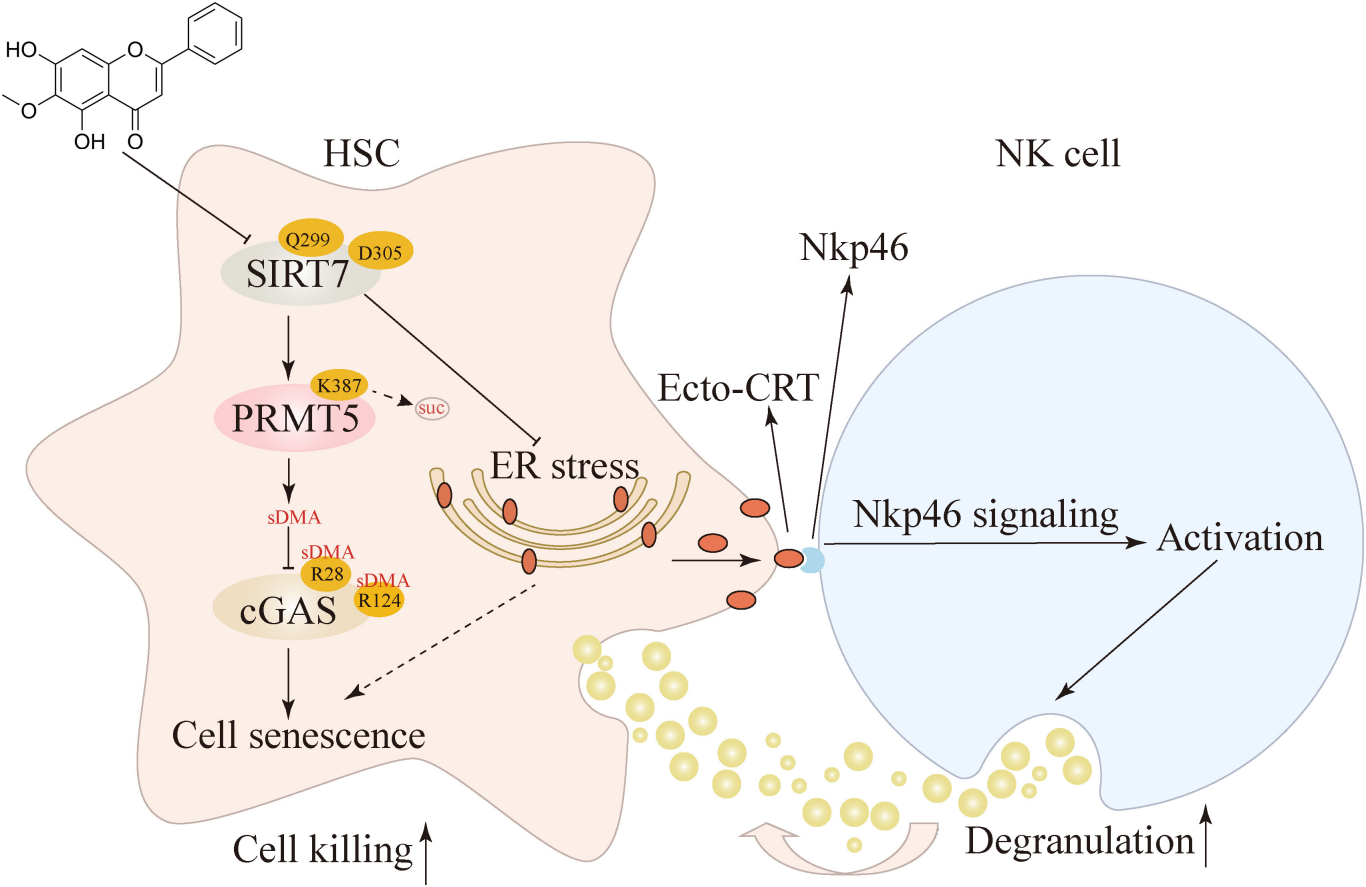
Mechanism diagram of the dual role of SIRT7 in OA inducing HSC senescence and promoting NK cell killing.

In conclusion, we show that SIRT7 in HSCs plays a dual role in the anti-hepatic fibrotic effects of OA, inhibiting HSC senescence via desuccinylase function and inhibiting NK cell killing via non-enzymatic function (Fig. 9). These findings will open up new possibilities for hepatic fibrosis detection and treatment.

## Materials and Methods

### Plasmids, antibodies, and reagents

The primary antibodies used are as follows: rabbit anti-cGAS (NBP3-12371, NOVUS), mouse anti-cGAS (SC-515802, Santa Cruz), rabbit anti-STING (A21051, ABclonal), rabbit anti-TBK1 (DF7026, AFFINITY), rabbit anti-p-TBK1 (AP0847, ABclonal), rabbit anti-IRF3 (A2172, ABclonal), rabbit anti-p-IRF3 (AP0857, ABclonal), rabbit anti-IL6 (A0286, ABclonal), rabbit anti-IL-1β (A20527, ABclonal), pan-symmetric di-methyl arginine rabbit pAb (A18261, ABclonal), mouse anti-succinyllysine mAb (PTM-419, PTM BIO), rabbit anti-PRMT5 (18436-1-AP, Proteintech), mouse anti-PRMT5 (SC-136202, Santa Cruz), rabbit anti-SIRT7 (29729-1-AP, Proteintech), mouse anti-SIRT7 (sc-365344, Santa Cruz), mouse anti-P21 (sc-397, Santa Cruz), rabbit anti-P16 (A0262, ABclonal), mouse anti-calregulin (SC-373863, Santa Cruz), rabbit anti-HA (51064-2-AP, Proteintech), DYKDDDDK tag monoclonal antibody (66008-4-Ig, Proteintech), rabbit anti-β-actin (AC026, ABclonal), and α-tubulin (11224-1-AP, Proteintech). Lipofectamine 3000 (Invitrogen Cat#L3000015) was used for the siRNA and plasmid transfections in accordance with the manufacturer’s recommendations. SIRT7-HA plasmid was constructed by General Biol Tech. pLV3-CMV-SIRT7 (human)-3*Flag, pLV3-CMV-SIRT7 (human)-R73A-3*Flag, pLV3-CMV-SIRT7 (human)-Q299A-3*Flag, pLV3-CMV-SIRT7 (human)-D305A-3*Flag, pLV3-CMV-SIRT7 (human)-Myc, pLV3-CMV-SIRT7 (human)-H187Y-Myc, PRMT5 &3*Flag plasmid, PRMT5 E444Q &3*Flag plasmid, PRMT5 K387R &3*Flag, and PRMT5 K387E &3*Flag. cGAS-HA plasmid, cGAS R28K HA plasmid, and cGAS R124K HA plasmid were constructed by EKBIOTech.

### Cell lines

For the human HSC line LX-2 (Cat. CL-0560) and human NK cell line NK-92mi (CL-0533), Procell Life Science & Technology provided the cells. Rat HSC line HSC-T6 was purchased from BDBIO (Hangzhou, China). HSC-T6 and LX2 cells were cultured as previously mentioned [12]. Human NK-92MI cells should be maintained in NK-92MI special medium (CM-0533). Mouse primary HSCs were isolated and cultured based on the well-established procedures [40,41].

### Patient specimens

We received human liver samples from patients in Nanjing Hospital Affiliated to Nanjing University of Chinese Medicine. Histologically staged fibrosis [no fibrosis (F0/1), *n* = 3], mild fibrosis (F2, *n* = 3), and severe fibrosis (F3/4, *n* = 6) were defined based on Metavir criteria. Previous articles described specific experimental procedures [41].

### The animal experiment design

Male ICR mice were housed in an air-conditioned room at 25 °C for a 12-h light/dark cycle. To generate a murine model of hepatic fibrosis, mice received intraperitoneal injections of 10% CCl_4_ (Sigma, St. Louis, Mo., USA) 3 times a week at a concentration of 0.5 μl/g for a duration of 8 weeks. Mice were given an injection of the HSC-specific VA-Lip-SIRT7 plasmid via the tail vein. All mice were shaken up at random and split up into 7 groups, *n* = 6 per group: control group, CCl_4_ model group, CCl_4_ +OA (40 mg/kg) group, CCl_4_+Vector group, CCl_4_+OA+vector group, CCl_4_+VA-Lip-SIRT7 plasmid group, and CCl_4_+OA+VA-Lip-SIRT7 plasmid group. Following an 8-week period, the mice were weighed, given a 2-min inhalation of isoflurane at a dosage of 1% to 1.5% to induce anesthesia, and blood was extracted from their orbits. The mouse livers were categorized and pictures were taken. After that, the livers were divided into 2 parts; paraformaldehyde was used to fix the first and the second was promptly refrigerated at −80 °C.

### Histological analysis

Previous articles described specific experimental procedures [12].

### Biochemical analysis

After 2 h of precipitation, mice’s ocular blood was centrifuged at 3,000 rpm to extract the serum. It was given to Nanjing Jinting Biotechnology Co., Ltd. to set up enzyme-linked immunosorbent assay (ELISA) analysis. Liver injury markers like ALT and ALP and hepatic fibrosis markers like HA and LN are measured using automated biochemistry (Hitachi, Ltd.; #7600P).

### Western blot

Previous articles described specific experimental procedures [12]. β-Actin, α-tubulin, and GAPDH were applied as the constant control.

### Human IFN-β ELISA kit

Using the Human IFN-β Elisa-Kit (MBE10166, MALLBI BIO), the amount of IFN-β generated was measured. Previous articles described specific experimental procedures [12].

### Human cGAMP ELISA kit

The amount of cGAMP present in LX2 cells was determined using the human cGAMP (cyclic GMP-AMP) ELISA kit (mlbio, Shang Hai, China). Previous articles described specific experimental procedures [12].

### Quantitative real time-PCR

Previous articles described specific experimental procedures [12]. Table S1 shows the primer sequences.

### Immunofluorescence assay

Utilize immunofluorescence to evaluate the expression of proteins in liver tissues, LX2 cells, and other cells. Previous articles described specific experimental procedures [12]. Cells were blocked with 5% goat serum without permeabilization in order to detect surface CRT. Mouse anti-calregulin (SC-373863, Santa Cruz) was used as the main antibody, and secondary antibodies (SA00007-1, Proteintech) were then applied. Utilizing a confocal imaging system with laser scanning (Leica TCS SP8), fluorescence pictures were captured and subjected to ImageJ analysis.

### Immunoprecipitation

Previous articles described specific experimental procedures [42].

### Molecular docking and molecular dynamics simulation

The SIRT7 protein structure was downloaded from the Alpha Fold Database [43,44]. Previous articles described specific experimental procedures [11].

### Cellular thermal shift assay

Briefly, LX2 cells were incubated with or without OA for 2 h, lysed applying cell lysates with RIPA buffer (P0013B, Beyotime) and protease inhibitors for 30 min. Afterwards, they were equally distributed into 10 parts. All parts were put under different temperature (50, 53, 56, 59, 62, 65, 68, 71, 74, and 77 °C) for 3 min. The level of SIRT7 was detected by WB.

### SA-β-gal activity assay

The SA-β-Gal Kit (C0602, Beyotime, China) was utilized to identify the senescence of LX2 cells following OA treatment. After the LX2 cells were fixed, enough staining solution was prepared per the instructions and the mixture was kept in an overnight incubator at 37 °C. The following day, the LX2 cells were examined with a standard optical microscope. Choose 3 fields of vision at random.

### Cytotoxicity assay

The cytotoxicity of NK cells against HSCs was assessed using the Crystal Violet Stain Solution (KGA229). Previous articles described specific experimental procedures [38].

### Transcriptome analysis

Mouse liver tissues were lysed using Trizol reagent. Concentrations were tested using Nanodrop2000, for the extracted nucleic acids, and integrity was tested using Agient2100, LabChip GX.

To produce single-stranded circular DNA, the duplex’s target region library underwent denaturation, cyclization, and digestion. The DNA Nano Ball (DNB) is the amplified product of the rolling circle amplification method, which is used to amplify single-stranded circular DNA. Load the prepared DNB onto the patterned array. For the sequencing, employ a combinatorial probe-anchor synthesis. Use a high-resolution imaging system to capture, read, and identify optical signals following the polymerization of the sequencing primer anchor molecules and fluorescent probes on DNA nanospheres in order to extract the single-base sequence data. Next, move on to the following cycle in order to acquire the subsequent base sequence data. After several cycles, raw sequencing data were eventually obtained.

### Bioinformatics analysis

This study used the GSE84044 dataset, which included liver tissue samples from 124 patients with chronic hepatitis B, focusing on exploring the gene expression profile of HBV-associated hepatic fibrosis. Samples were measured by row gene expression from the Affymetrix Human Genome U133 Plus 2.0 microarray platform. First, differential gene analysis was used to identify genes significantly expressed in different stages of fibrosis, and gene modules associated with fibrosis progression, such as blue DEGs modules, were identified by weighted gene co-expression network analysis. Then gene set enrichment analysis (GSEA) was used to further reveal the expression of differential genes in the progression of hepatic fibrosis.

### Single-cell transcriptome sequencing analysis

This study used scLiverDB (http://bioinfo.life.hust.edu.cn/liverdb) for single-cell transcriptome analysis of the GSE137720 and GSE136103 datasets from the GEO database and visualized genes’ average expression and the percentage of cells expressing in different cell types (dot plot) [30]. Meanwhile, KEGG enrichment was analyzed for UP DEGs in NK cells.

### Statistical analysis

The experimental data are presented as mean ± standard error of the mean. To ascertain whether the normal distribution data were significantly different (comparison between the 2 groups), the Student *t* test was employed. For all statistical comparisons between groups, GraphPad Prism 8.0 (San Diego, California, USA) was utilized to conduct one-way analysis of variance, which was followed by the Tukey multiple comparison test. Statistical significance was considered where *P* < 0.05.

## Data Availability

The data are available from the corresponding authors on reasonable request.
