# Supplementary material for: Dual Roles of SIRT7 Inhibition by Oroxylin A Reprogram HSCs Fate: PRMT5 Succinylation-Driven Senescence and Ecto-Calreticulin-Dependent NK Cell Immune Clearance in Liver Fibrosis
Source: Research (Wash D C). 2025 Aug 7;8:0808. doi: 10.34133/research.0808 (PMC12329212; doi:10.34133/research.0808)
Supplement: Supplementary 1 — Figs. S1 to S3 Tables S1 and S2 [file research.0808.f1.zip › SUPPLEMENTARY MATERIALS.docx]

SUPPLEMENTARY MATERIALS

**Supplementary Table 1** Primer Sequences used for qRT-PCR.

| Genes | Forward primer | Reverse primer |
| --- | --- | --- |
| Human |  |  |
| IL-6 | CCTGAACCTTCCAAAGATGGC | TTCACCAGGCAAGTCTCCTCA |
| STING | GGGCTGAAGTAGAGTGGCACAATC | TGGGAGGCTAAGGCAGGAGAATC |
| TBK1 | TGCACCCTGATATGTATGAGAGA | AAATGGCAGTGATCCAGTAGC |
| IRF3 | AGAGGCTCGTGATGGTCAAG | AGGTCCACAGTATTCTCCAGG |
| SIRT7 | CGTTAGTGCTGCCGACCTGAG | GTGGAGCCCGTCACAGTTCTG |
| GAPDH | CCAACCGCGAGAAGATGA | CCAGAGGCGTACAGGGATAG |
| Mouse |  |  |
| SIRT7 | CCTGCATCCCTAACAGAGAG | AGCTGGACCCTAAACACAGG |

**Supplementary Table 2** A compilation of data about energy, pressure, density, and further simulation-related changes.

| GROMACS Energies | |
| --- | --- |
| LJ (SR)(kJ·mol^-1^) | 2047744.65±1245.56 |
| Coulomb (SR)(kJ·mol^-1^) | -1661649.53±2000.78 |
| Potential(kJ·mol^-1^) | -1342339.82±1303.91 |
| Kinetic En. (kJ·mol^-1^) | 248513.21±860.77 |
| Total Energy(kJ·mol^-1^) | -1093826.612±1691.16 |
| Temperature(K) | 298.15±1.03 |
| Pressure(bar) | 1.129±84.16 |
| Density(kg·m^-3^) | 994.66±1.57 |

**Supplementary fig. 1**

**Supplementary fig. 1** OA reduced the protein expression of SIRT7 and PRMT5 in HSCs. (A-B,F-G) WB analyzed the protein expression of PRMT5 and SIRT7 in HSCs. (C) IP analysis showed the interaction between cGAS and PRMT5. (D) Acetylation level of PRMT5 was tested by IP analysis treated with OA or DMSO. (E) Immunofluorescence screening was employed to detect the protein expression and colocalization of α-SMA and SIRT7. The data are shown as mean ± SD (n=3-5). *P < 0.05, **P < 0.01, ***P < 0.001 vs control group. ^&^P < 0.05 vs Model-4 weeks. ^%%^P < 0.01 vs Model-8 weeks.

**Supplementary fig. 2**

**Supplementary fig. 2** OA significantly inhibited liver fibrosis in HSCs. (A-E) WB analyzed the protein expression of PRMT5, α-SMA, collagen I, fibronectin and HGF in HSCs. (F) IP analysis showed the interaction between SIRT7 and PRMT5. The data are shown as mean ± SD (n=3). *P < 0.05, **P < 0.01, ***P < 0.001 vs control group. ns, no siginificant; ^#^P < 0.05, ^##^P < 0.01, ^###^P < 0.001 vs OA group.

**Supplementary fig.3**

**Supplementary fig. 3** OA inhibiting SIRT7 decreases PRMT5 expression, thus triggering the cGAS-STING pathway. (A) Immunofluorescence screening was applied to shown the protein expression α-SMA. (B-D) Immunofluorescence screening was employed to detect the protein expression and colocalization of α-SMA and cGAS (B), α-SMA and STING (C), α-SMA and PRMT5(D). The colocalization were quantified with Image J software. 100 µm scale bars are used. n = 6 in every group, *P < 0.05, **P < 0.01, ***P < 0.001 vs control group; ^#^P < 0.05, ^##^P < 0.01, ^###^P < 0.001 vs CCl_4_+Vector group and ns, no significant, ^&&^P < 0.01, ^&&&^P < 0.001 vs CCl_4_+OA+Vector group).
