# Supplementary figures and images for: Dual Roles of SIRT7 Inhibition by Oroxylin A Reprogram HSCs Fate: PRMT5 Succinylation-Driven Senescence and Ecto-Calreticulin-Dependent NK Cell Immune Clearance in Liver Fibrosis
Source: Research (Wash D C). 2025 Aug 7;8:0808. doi: 10.34133/research.0808 (PMC12329212; doi:10.34133/research.0808)

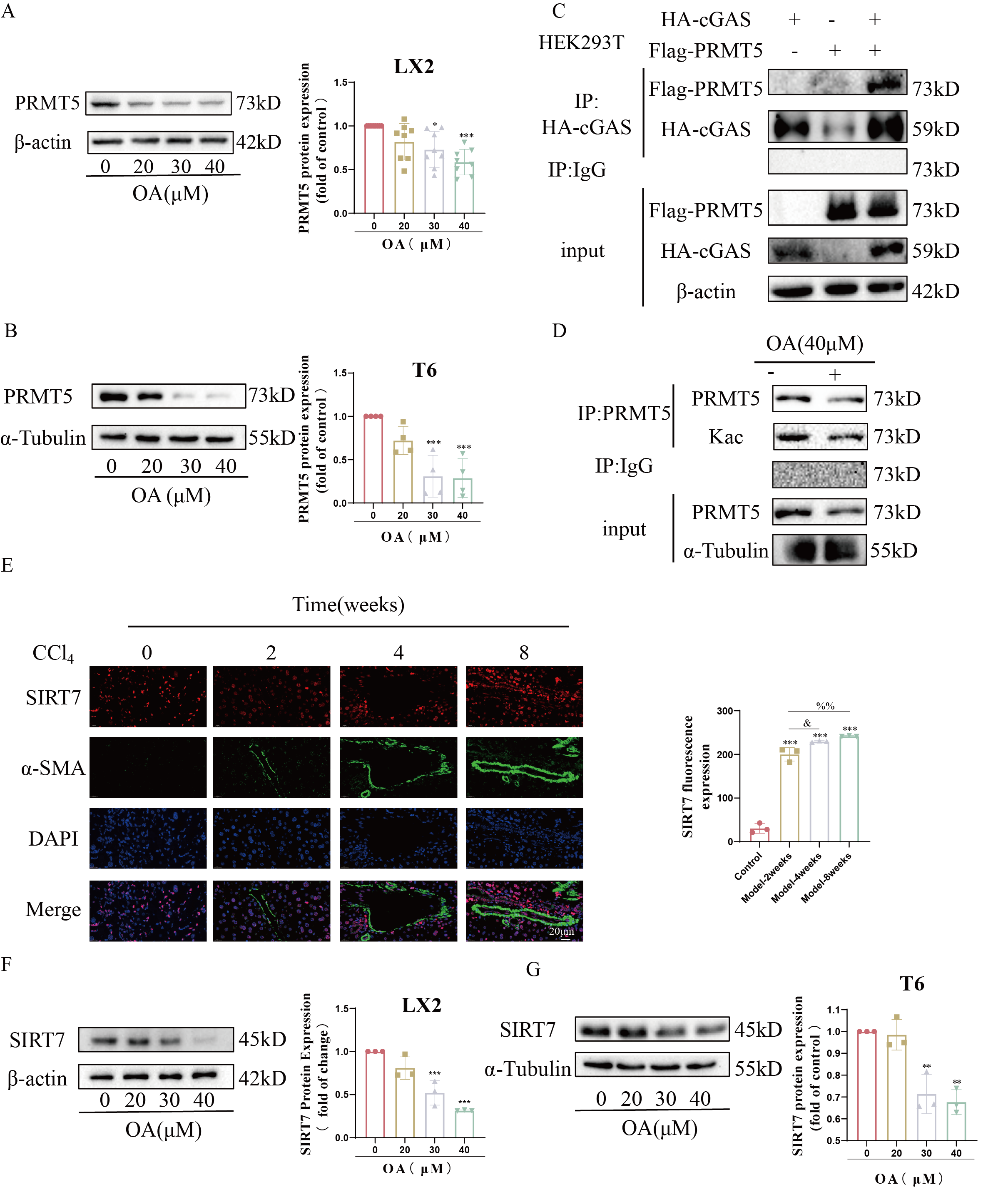

Supplement: Supplementary 1 — Figs. S1 to S3 Tables S1 and S2 [file research.0808.f1.zip › Supplementary Fig. 1.tif]

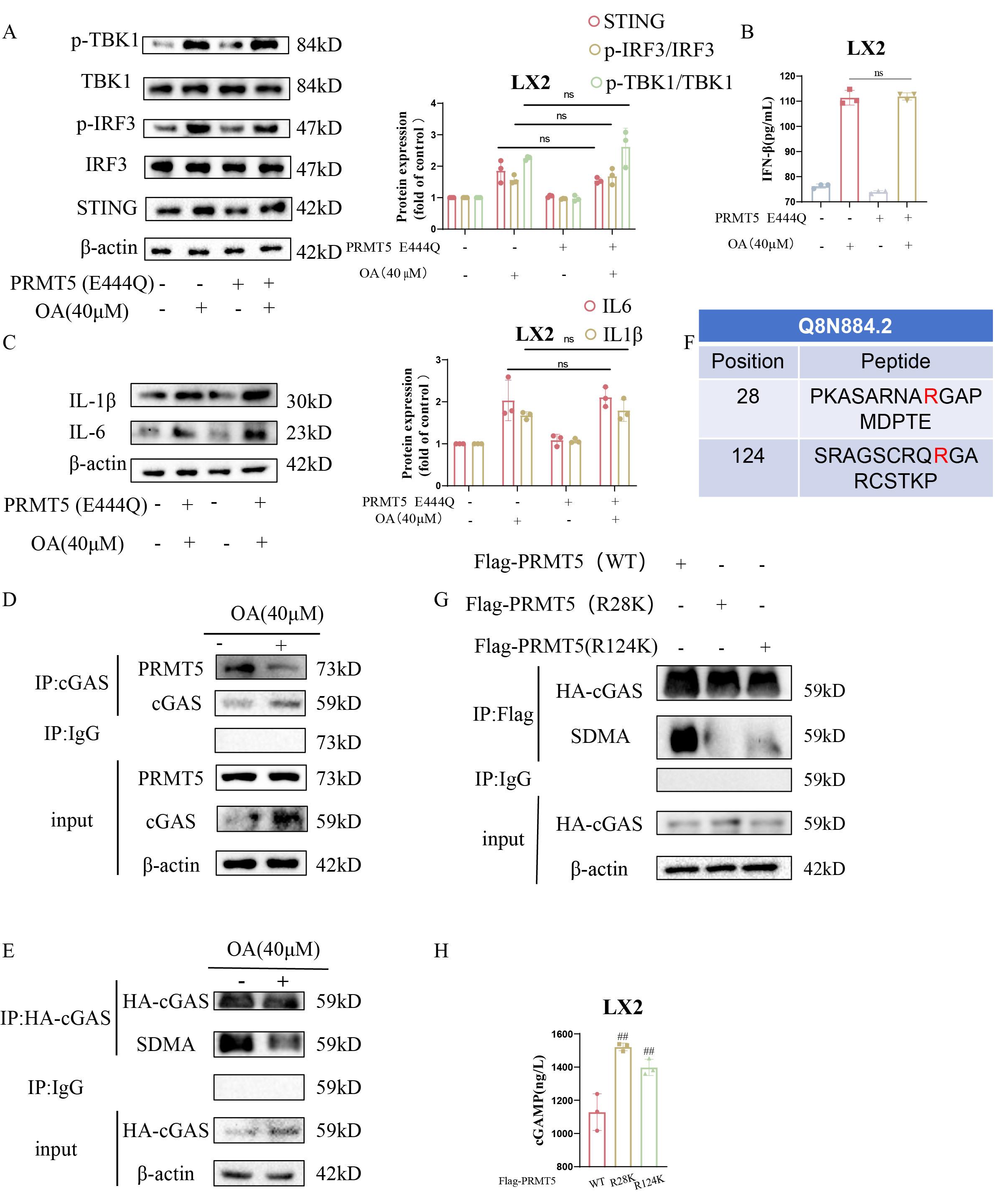

Supplement: Supplementary 1 — Figs. S1 to S3 Tables S1 and S2 [file research.0808.f1.zip › Supplementary Fig. 2.tif]

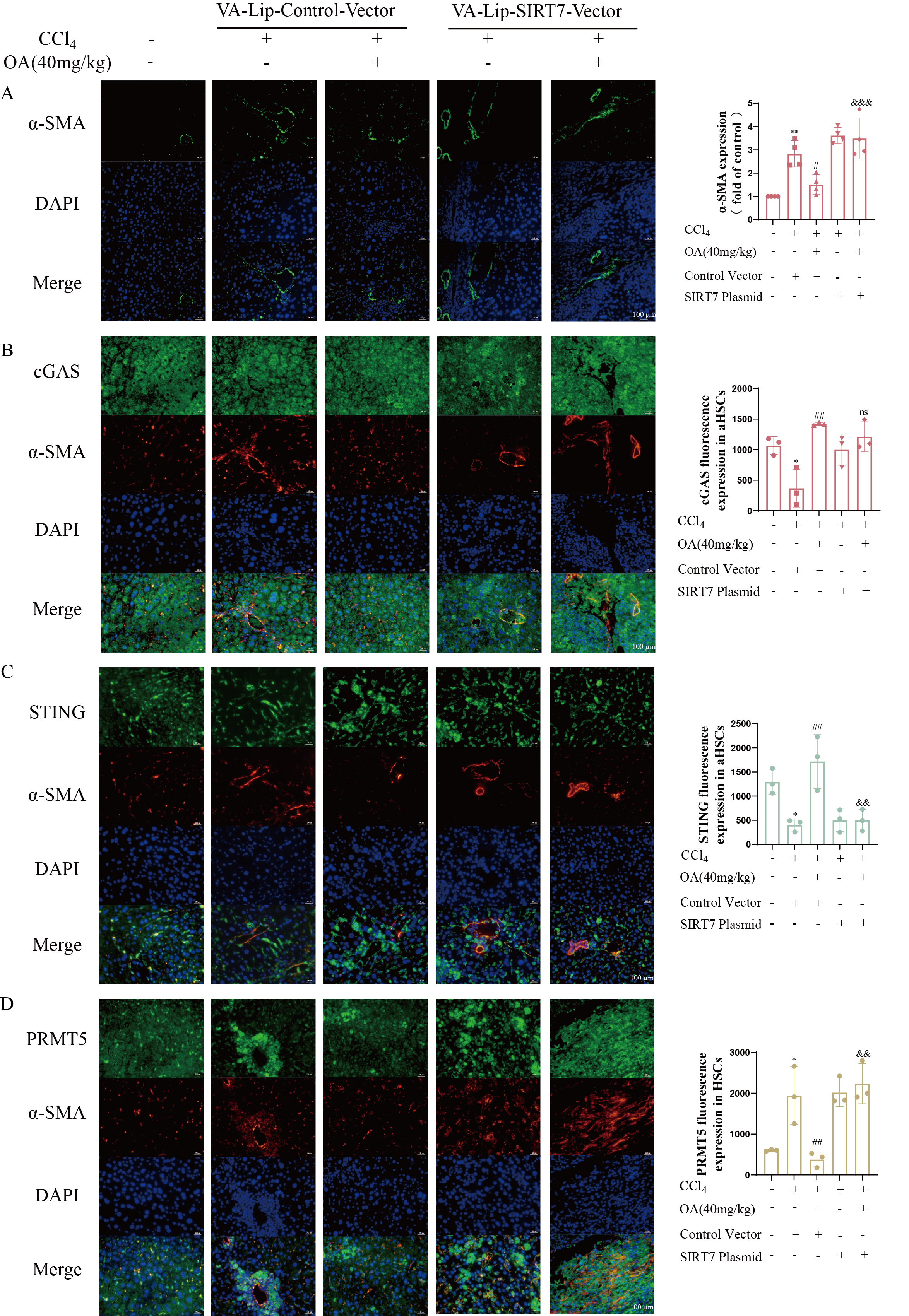

Supplement: Supplementary 1 — Figs. S1 to S3 Tables S1 and S2 [file research.0808.f1.zip › Supplementary Fig. 3.tif]

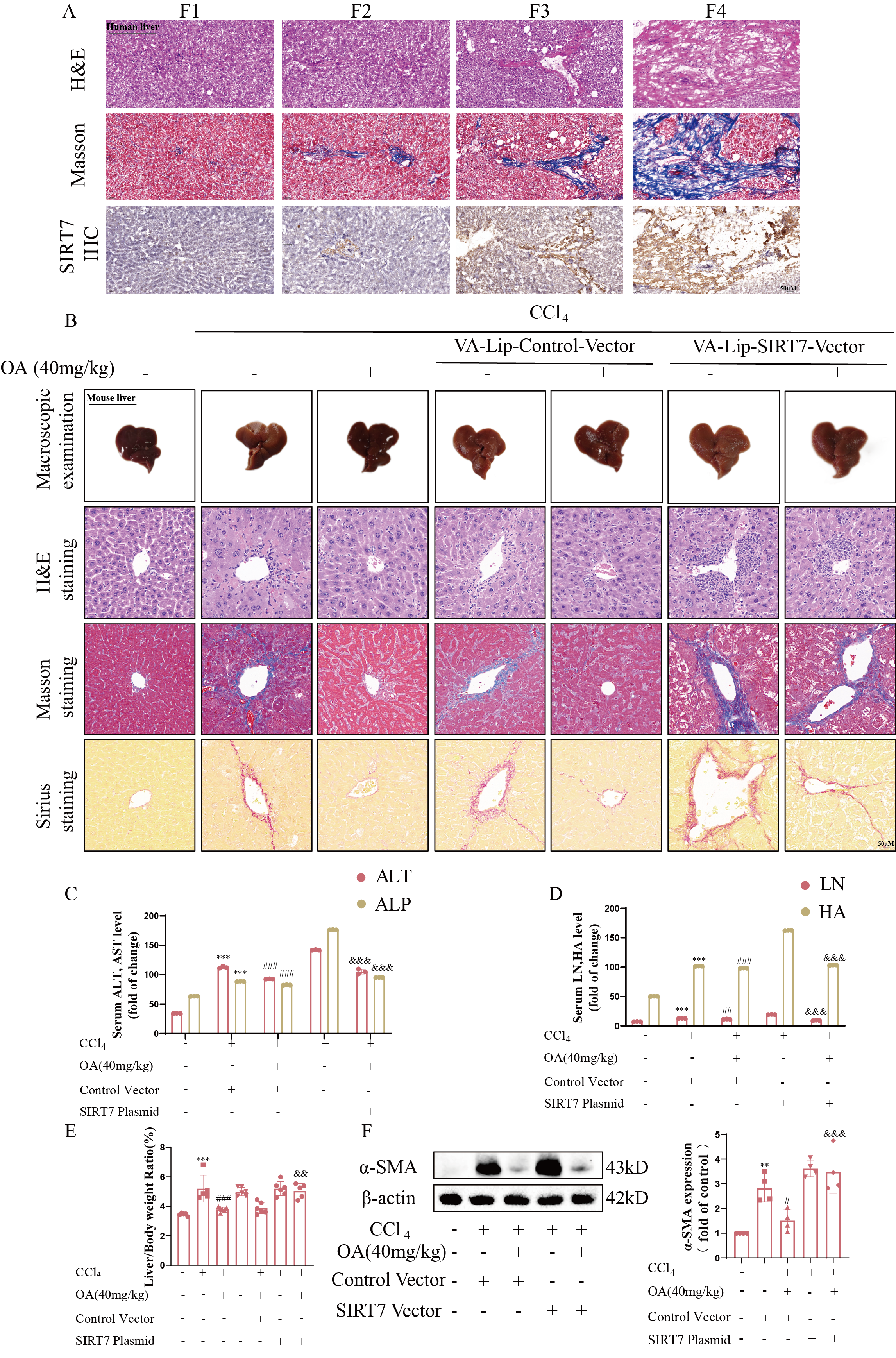

Supplement: Supplementary 1 — Figs. S1 to S3 Tables S1 and S2 [file research.0808.f1.zip › Supplementary Fig. 6.tif]

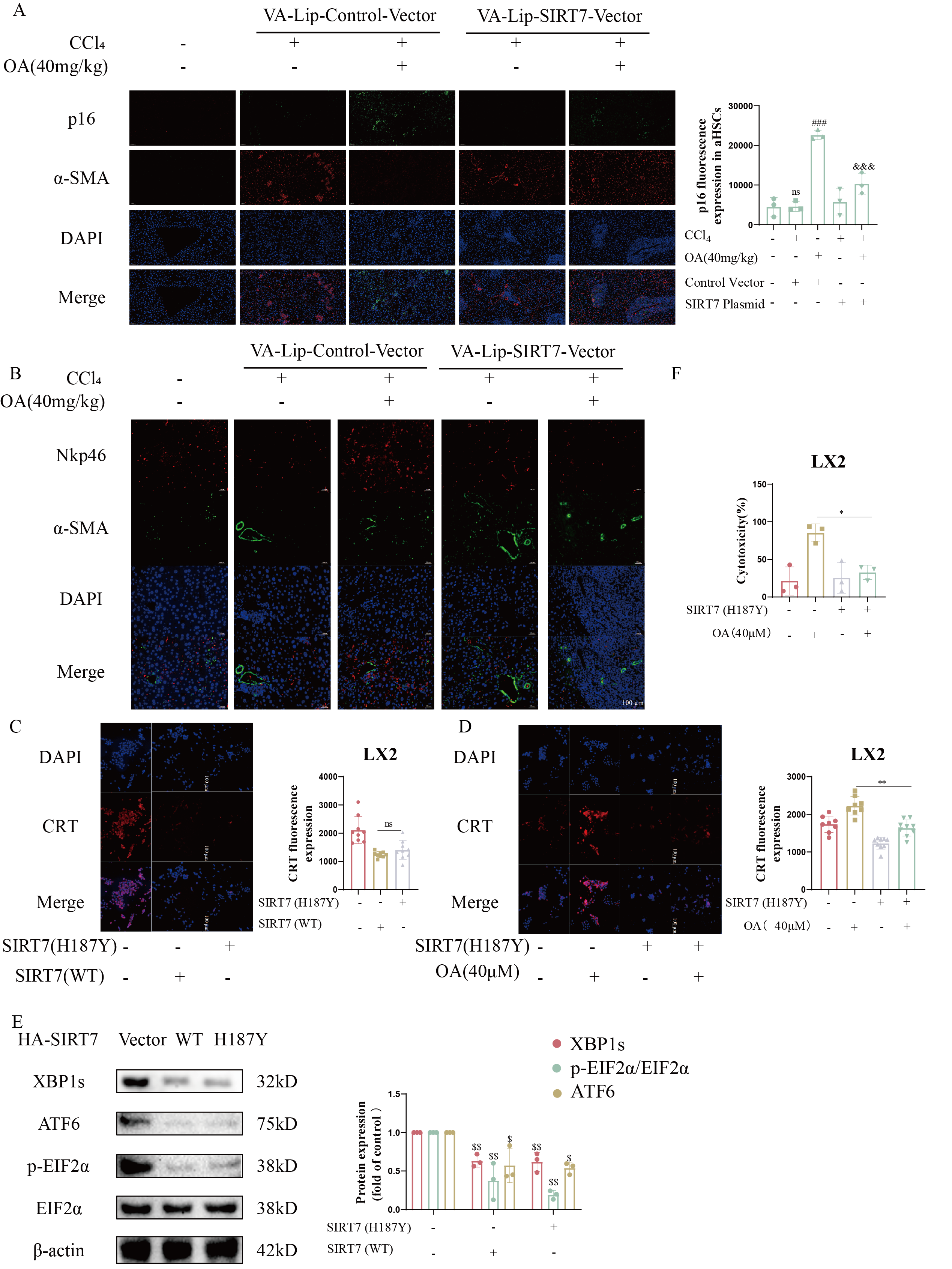

Supplement: Supplementary 1 — Figs. S1 to S3 Tables S1 and S2 [file research.0808.f1.zip › Supplementary Fig. 8.tif]
